# [Corrigendum] Overexpression of microRNA-96-5p inhibits autophagy and apoptosis and enhances the proliferation, migration and invasiveness of human breast cancer cells

**DOI:** 10.3892/ol.2026.15897

**Published:** 2026-10-08

**Authors:** Yawei Shi, Yang Zhao, Nan Shao, Runyi Ye, Yin Lin, Ning Zhang, Wen Li, Yunjian Zhang, Shenming Wang

Oncol Lett 13: 4402-4412, 2017; DOI: 10.3892/ol.2017.6025

Subsequently to the publication of the above paper, an interested reader drew to the authors’ attention that, concerning the Transwell cell migration and invasion assay data shown in Figs. 7D and 8A on p. 4408 and 4409 respectively, the panel furthest on the left in Fig. 7D shared an overlapping section of data with the “MCF-7/MiR-96-5 mimic” data panel in Fig. 8A, such that these data appeared to have been derived from the same original source where different experimental conditions were intended to have been shown. Additionally, upon performing an independent analysis of the data in this paper in the Editorial Office, it came to light that there were apparently three protein bands held in common comparing the Ac-FoxO-1 data for the MDA-MB-231 cell line (Fig. 6A, upper right panels) with the three rightmost protein bands for the Ac-FoxO-1 data for the MCF-7 cell line (bottom left panels) in Fig. 6B.

The authors were able to re-examine their original data, and realize that the data included in Figs. 7D and 6A were inadvertently selected incorrectly. The revised versions of Figs. 6 and 7, now showing the correct data for the panel furthest on the left in Fig. 7D and the Ac-FoxO-1 data for the MDA-MB-231 cell line in Fig. 6A, are shown on the next two pages. The authors regret the errors that were made while compiling the original figures, and are thankful to the editor of *Oncology Letters* for allowing them the opportunity to publish this Corrigendum. Note that the errors that were made in this pair of figures did not have a significant impact on the conclusions reached in this study. All the authors agree with the publication of this corrigendum; furthermore, they apologize to the readership for any inconvenience caused.

## Figures and Tables

**Figure 6.**
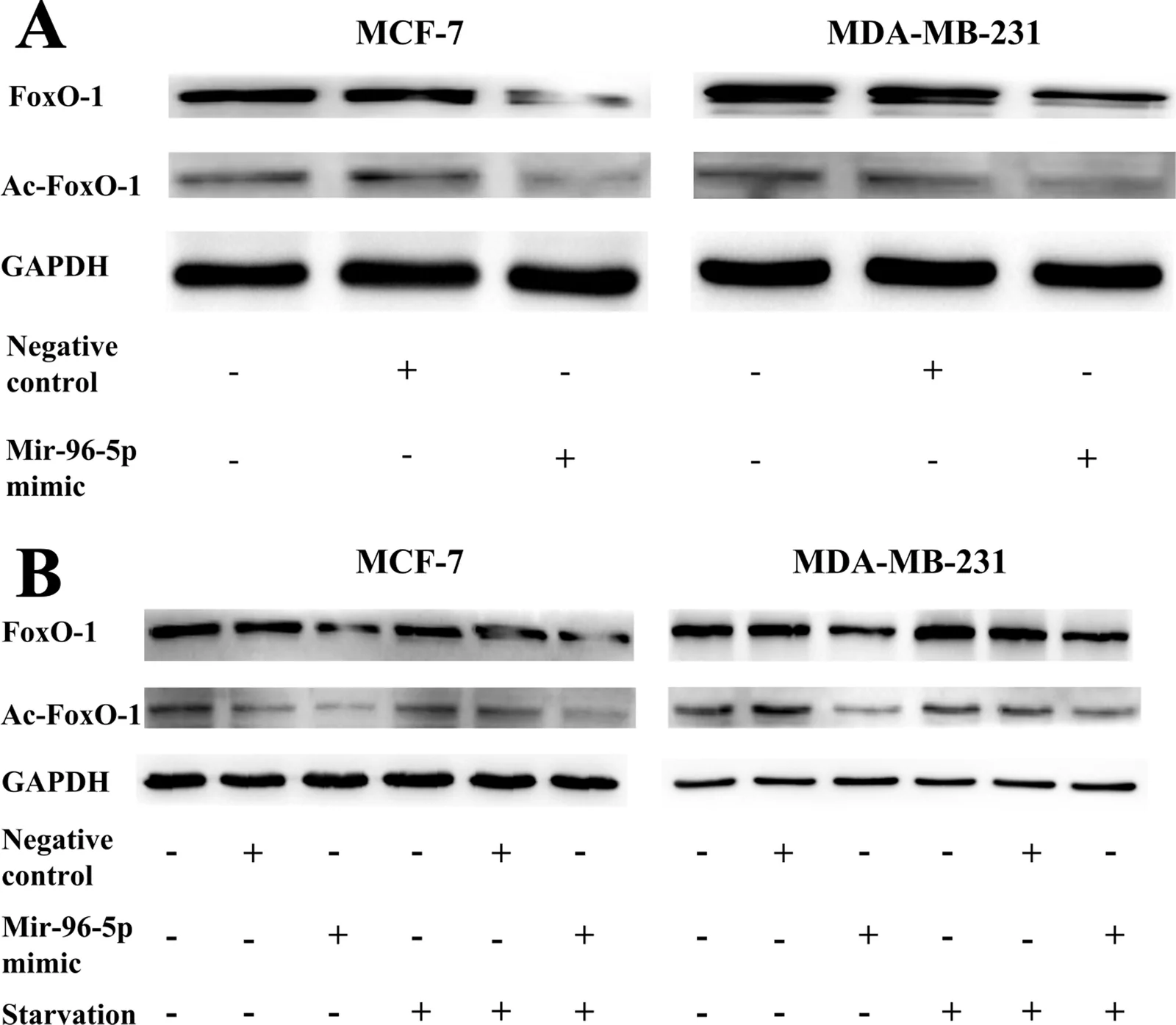
miR-96-5p decreased FOXO1 and Ac-FOXO1 expression levels in MCF-7 and MDA-MB-231 cells. (A) MCF-7 and MDA-MB-231 cells were transfected with a miR-96-5p mimic or a control construct. Western blotting demonstrated that FOXO1 and Ac-FOXO1 protein expression was decreased with miR-96-5p overexpression. (B) MCF-7 and MDA-MB-231 cells were transfected with a miR-96-5p mimic or a control construct and then treated with EBSS for 4 h. Western blotting illustrated that FOXO1 and Ac-FOXO1 levels were downregulated in the miR-96-5p overexpression groups, but no significant difference was observed between the non-starved and starved cells. GAPDH was used as the loading control. The results are representative of three independent experiments (mean ± standard deviation of independent experiments, n=3). FOXO1, forkhead box protein O1; Ac-FOXO1, acetylated-FOXO1; miR, microRNA.

**Figure 7.**
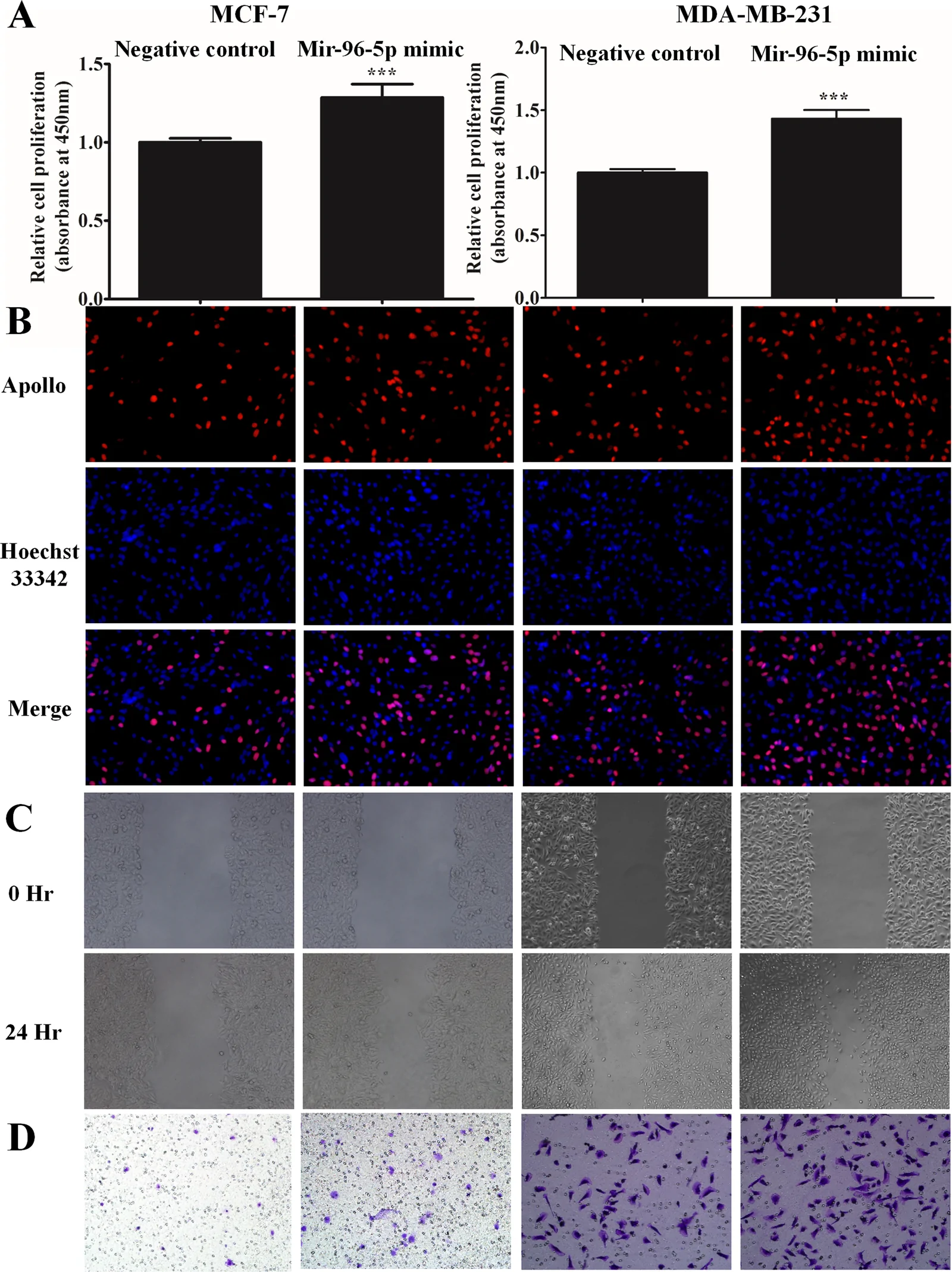
The overexpression of miR-96-5p promoted the proliferation and migration of MCF-7 and MDA-MB-231 cells. First, the cells were transfected with 50 nmol/l miR-96-5p mimic or a control construct. (A and B) The proliferation of the MCF-7 and MDA-MB-231 cells was determined using (A) a Cell Counting kit-8 assay and (B) an EdU assay. (C and D) Cell migration was evaluated by (C) a scratch assay and (D) a Transwell assay at 0 and 24 h time points (***P<0.001). Hoechst 33342, bisbenzimide; miR, microRNA; EdU, 5-ethynyl-2’-deoxyuridine.

